# Trends in the likelihood of receiving percutaneous coronary intervention in a low-volume hospital and disparities by sociodemographic communities

**DOI:** 10.1371/journal.pone.0279905

**Published:** 2023-01-18

**Authors:** Christina Wang, Karla Lindquist, Harlan Krumholz, Renee Y. Hsia

**Affiliations:** 1 Department of Medicine, University of California San Francisco, San Francisco, California, United States of America; 2 Department of Epidemiology and Biostatistics, University of California San Francisco, San Francisco, California, United States of America; 3 Department of Cardiovascular Medicine, Yale School of Medicine, New Haven, Connecticut, United States of America; 4 Department of Emergency Medicine, University of California San Francisco, San Francisco, California, United States of America; 5 Philip R. Lee Institute for Health Policy Studies, University of California San Francisco, San Francisco, California, United States of America; The University of Mississippi Medical Center, UNITED STATES

## Abstract

**Introduction:**

Over the past two decades, percutaneous coronary intervention (PCI) capacity has increased while coronary artery disease has decreased, potentially lowering per-hospital PCI volumes, which is associated with less favorable patient outcomes. Trends in the likelihood of receiving PCI in a low-volume center have not been well-documented, and it is unknown whether certain socioeconomic factors are associated with a greater risk of PCI in a low-volume facility. Our study aims to determine the likelihood of being treated in a low-volume PCI center over time and if this likelihood differs by sociodemographic factors.

**Methods:**

We conducted a retrospective cohort study of 374,066 hospitalized patients in California receiving PCI from January 1, 2010, to December 31, 2018. Our primary outcome was the likelihood of PCI discharges at a low-volume hospital (<150 PCI/year), and secondary outcomes included whether this likelihood varied across different sociodemographic groups and across low-volume hospitals stratified by high or low ZIP code median income.

**Results:**

The proportion of PCI discharges from low-volume hospitals increased from 5.4% to 11.0% over the study period. Patients of all sociodemographic groups considered were more likely to visit low-volume hospitals over time (P<0.001). Latinx patients were more likely to receive PCI at a low-volume hospital compared with non-Latinx White in 2010 with a 166% higher gap in 2018 (unadjusted proportions). The gaps in relative risk (RR) between Black, Latinx and Asian patients versus non-Latinx white increased over time, whereas the gap between private versus public/no insurance, and high versus low income decreased (interaction P<0.001). In low-income ZIP codes, patients with Medicaid were less likely to visit low-volume hospitals than patients with private insurance in 2010; however, this gap reversed and increased by 500% in 2018. Patients with low income were more likely to receive PCI at low-volume hospitals relative to patients with high income in all study years.

**Conclusions:**

The likelihood of receiving PCI at low-volume hospitals has increased across all race/ethnicity, insurance, and income groups over time; however, this increase has not occurred evenly across all sociodemographic groups.

## Introduction

Many studies have documented the association between higher operator or hospital percutaneous coronary intervention (PCI) volume and improved patient outcomes, including inpatient mortality [1], 30-day mortality, myocardial infarction (MI), repeat vascularization, and the need for urgent coronary artery bypass grafting surgery [2]. The American College of Cardiology Foundation/American Heart Association Task Force on Practice Guidelines and the Society for Cardiovascular Angiography and Interventions (ACCF/AHA/SCAI) performed a systematic review which found that a per-hospital volume threshold of <200 PCIs/year is consistently associated with higher incidence of inpatient mortality and adverse events [3]. These guidelines are often used in policies governing PCI hospital certification, including eligibility to perform PCI without on-site cardiac surgery, according to California Department of Public Health regulations [4, 5].

From 2003–2016, total per capita PCI volume in the United States decreased by 43% [6], resulting in lower per hospital PCI volumes [7–9]. This raises the question: how do changes in PCI procedural volumes influence the likelihood that a patient will receive PCI at a low-volume hospital? Further, sociodemographic disparities in cardiovascular care and outcomes are well established by race/ethnicity, insurance type, and income [10–18], and evidence suggests that disparities in PCI outcomes may be increasing [10, 17]. Our primary objective was to determine the likelihood of being treated in a low-volume PCI center over time and describe the sociodemographic characteristics of patients treated in these centers. Our secondary objective was to measure this likelihood across different sociodemographic groups and stratify results by hospital ZIP code median income. We hypothesized that Black, Latinx patients, patients with Medicaid, and low-income patients would be increasingly more likely to receive PCI at low-volume hospitals relative to non-Latinx White, privately insured, and high-income patients, respectively.

## Methods

### Data

We used nonpublic patient data from the California Office of Statewide Health Planning and Development (OSHPD) [19]. This database includes discharges from every inpatient encounter from every acute care hospital in California (excluding Veterans Affairs and military facilities). This study was approved by the Committee for Human Research of our institution.

### Population

We included all discharges of adults (≥18 years of age) from January 1, 2010, to December 31, 2018, who received PCI. Prior to October 1, 2015, we analyzed procedure codes from the International Classification of Diseases 9^th^ Revision Clinical Modification (ICD-9-CM) for transluminal coronary atherectomy, percutaneous transluminal coronary angioplasty, insertion of non-drug-eluting coronary artery stent(s), and insertion of drug-eluting coronary stent(s), as previously validated in other datasets and analyses (S1 Table) [6, 20, 21]. For dates after October 1, 2015, we used codes from the ICD 10^th^ Revision (ICD-10) Procedure Coding System (PCS) for percutaneous coronary and endoscopy artery dilations, consistent with PCI codes used by the OSHPD Healthcare Analytics Branch, the Agency for Healthcare Research and Quality, and previous studies (S2 Table) [6, 22, 23]. All comorbidities were identified in a similar manner using codes outlined in S3 Table. Myocardial infarction was considered a comorbidity only if it was the principal diagnosis associated with the patient’s hospitalization. Entries with missing data were included in the total PCI volume count. Race/ethnicity and payer variables populated as “missing” or “unknown” were categorized as “other”. ZIP codes without income data were omitted from model results.

### Variables

We extracted patient-level data for age, sex, race/ethnicity, insurance, and all other clinical diagnoses and procedural codes incurred during admission from OSHPD. We identified PCI-capable hospitals by any “oshpd_id” associated with at least one procedural or diagnostic code for PCI as described above. We categorized race and ethnicity using the OSHPD “race_grp” categories of non-Latinx White, Black, Asian, and Latinx, with all other categories defined as “other.” Of note, OSHPD data automatically categorizes anyone selecting “Hispanic” ethnicity, which is a separate variable, as “Hispanic” under the “race_grp” variable. We grouped payers as Medicare, Medicaid, private, and self-pay, with all other categories defined as “other.” We categorized every visit as low-income (<25th percentile), medium-income (25-75th percentile), and high-income (>75th percentile) within California ZIP codes (90000 to 96162, inclusive) [24] by linking the patient or hospital ZIP code to the corresponding median household income for that ZIP code based on the 2018 American Community Survey 5-year estimates from Census data [25].

Consistent with previous literature, we accounted for expected outpatient PCI volume using inpatient PCI volume as a proxy [26], we approximated that two-thirds of an institution’s PCIs performed would qualify for inpatient PCI [27, 28]. We classified PCI hospitals as low-volume (≤150) or high-volume (>150) for the absolute number of inpatient PCIs performed in a given year. This threshold was based on the 200 total PCI/year threshold suggested by ACCF guidelines [5], as well as reported associations between <150 PCIs/hospital per year and higher in-hospital mortality and rates of periprocedural complications [29].

### Statistical approach

Our primary outcome was the proportion of annual discharges with a PCI procedure from hospitals performing fewer or equal to 150 inpatient PCI / year. To assess any statistically significant differences between groups (e.g. Table 1), t-tests were used between groups with continuous variables and chi-square tests were used between groups with categorical variables. To assess the statistical significance of trends in visiting low-volume hospitals over the study period, we ran a logistic regression model including as continuous covariates year, age, and number of visits over the entire observation period, and as categorical covariates sex, race/ethnicity, insurance type, patient income, and comorbidities. Comorbidities included MI, cardiogenic shock, congestive heart failure, cerebrovascular accident, diabetes, hypertension, and chronic kidney disease. For the regression model, we used only records with a linkage number identifying the unique patient (>92% of all observations).

**Table 1:** Baseline demographics of patients receiving PCI (2010–18) (N = 374,066).

Variable	All Discharges	Low-Volume	High-Volume	P-Value
PATIENT CHARACTERISTICS				
Age	Median: 66	Median: 64	Median: 66	<0.001
	[57–75]	[56–74]	[57–75]	
Male	245,978 (69.5%)	22,071 (69.9%)	223,907 (69.4%)	0.0465
**Race/Ethnicity**				
Latinx	71,447 (20.2%)	7,300 (23.1%)	64,147 (19.9%)	<0.001
Non-Latinx White	203,749 (57.5%)	16,164 (51.2%)	187,585 (58.1%)	
Black	19,879 (5.6%)	2,260 (7.2%)	17,619 (5.5%)	
Asian	35,614 (10.1%)	3,780 (12.0%)	31,834 (9.9%)	
Native American	868 (0.3%)	116 (0.4%)	752 (0.2%)	
Other	22,643 (6.4%)	1,937 (6.1%)	20,706 (6.4%)	
**Insurance**				
Medicare	181,756 (51.3%)	14,578 (46.2%)	167,178 (51.8%)	<0.001
Medicaid	46,001 (13.0%)	5,086 (16.1%)	40,915 (12.7%)	
Private	102,420 (28.9%)	9,521 (30.2%)	92,899 (28.8%)	
Self-pay	10,331 (2.9%)	1,107 (3.5%)	9,224 (2.9%)	
Other	13,692 (3.9%)	1,265 (4.0%)	12,427 (3.9%)	
CLINICAL CHARACTERISTICS				
Principal diagnosis of MI	204,513 (57.7% of discharges)	20,734 (65.7% of discharges)	183,779 (57.0% of discharges)	<0.001
Cardiogenic shock	25,998 (7.3%)	2,507 (7.9%)	23,491 (7.3%)	<0.001
CHF	85,744 (24.2%)	7,813 (24.8%)	77,931 (24.2%)	0.017
CVA	9,775 (2.8%)	839 (2.7%)	8,936 (2.8%)	0.257
Diabetes	143,512 (40.5%)	130,821 (40.6%)	12,691 (40.2%)	0.256
Hypertension	233,957 (66.1%)	19,669 (62.3%)	214,288 (66.4%)	<0.001
CKD	69,737 (19.7%)	6,165 (19.5%)	63,572 (19.7%)	0.481
HOSPITAL CHARACTERISTICS				
**Income Patterns**				
Income computed using hospital’s ZIP code	Median: 70,106	Median: 73,985	Median: 69,824	<0.001
	[56,239–95,852]	[53,615–91,947]	[56,239–95,852]	
Income computed using patients’ ZIP code	Median: 67,970	71,776	67,772	<0.001
	[52,096–90,906]	[53,615–96,812]	[51,899–90,172]	
**Yearly Volume**				
All-cause discharges regardless of PCI during admission	Median: 16,819	Median: 9,862	Median: 17,226	<0.001
	[11,432–21,652]	[7,581–14,190]	[12,917–21,771]	

To assess the temporal trend of the proportion of all PCI at low-volume hospitals by sociodemographic group, we ran a logistic regression model which included an interaction between year and race/ethnicity, insurance type, and sociodemographic subgroup. Non-Latinx white race/ethnicity, private insurance, and low income were used as the reference groups for the respective sociodemographic categories.

We used Huber-White robust standard errors in all models and corrected for multiple comparisons given that subsets of outcomes were analyzed in separate models using Holm’s method [30]. A P<0.05 family-wise error rate was considered statistically significant. Odds ratios and their 95% confidence intervals were converted to risk ratios (RRs) using a simple method based on the prevalence of the uncommon outcomes [31]. We performed all analyses using SAS 8.4 (SAS Institute Inc., Cary, North Carolina) and R version 4.0.2 (R Core Team. R: A Language and Environment for Statistical Computing. R Foundation for Statistical Computing, Vienna, Austria, 2020. https://www.R-project.org/). This project is considered IRB exempt.

## Results

**Fig 1:**
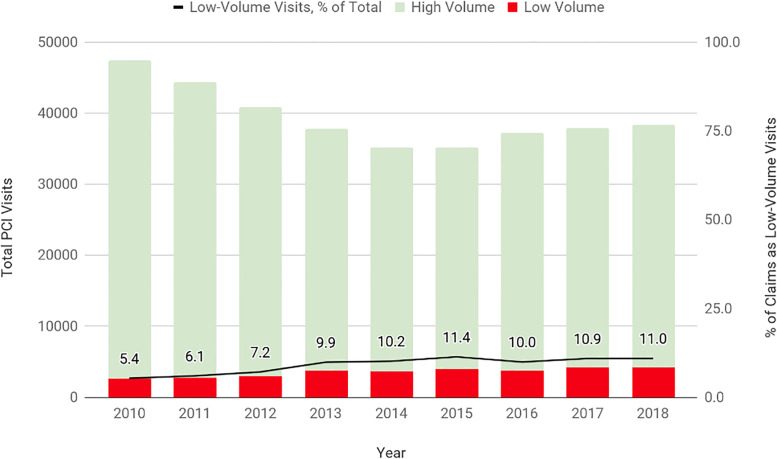
Total PCI volume by year and hospital volume (2010–2018).

From 2010 to 2018, the number of inpatient PCIs performed decreased from 47,458 in 2010 to 38,307 in 2018 (Fig 1). The total number of hospitals performing PCI in the dataset increased by 22 hospitals across 9 years, from 153 in 2010 to 175 in 2018. The proportion of total visits at low-volume hospitals grew from 5.4% in 2010 to 11.0% in 2018.

A total of 374,066 patients received an in-hospital PCI procedure between January 1, 2010, and December 31, 2018 (Table 1). The median age at admission was 66 years (range: 57–75 years), and most patients were male (69.5%). Non-Latinx White was the majority racial/ethnic group (57.5%), and a larger share of these patients were treated at high-volume hospitals as opposed to low-volume hospitals (58.1% vs. 51.2%). Most visits across the entire cohort were covered by Medicare (51.3%). Patients seen at low-volume hospitals were more likely to present with a principal discharge diagnosis of acute myocardial infarction (65.7% vs. 57.0%) and more likely to present with cardiogenic shock (7.9% vs. 7.3%) (all P<0.001).

Low-volume hospitals had a higher median ZIP code income ($73,985) than high-volume hospitals ($69,824) (P<0.001); see Table 1. The same trend was seen in median patient income for visits in low-volume ($71,776) vs. high-volume ($67,772) PCI hospitals (P<0.001). The median number of all-cause discharges was 9,862 per year for low-volume PCI hospitals and 17,226 per year for high-volume PCI hospitals (P<0.001).

### Likelihood of PCI at low-volume hospital in 2010 versus 2018

#### By race/ethnicity

**Fig 2:**
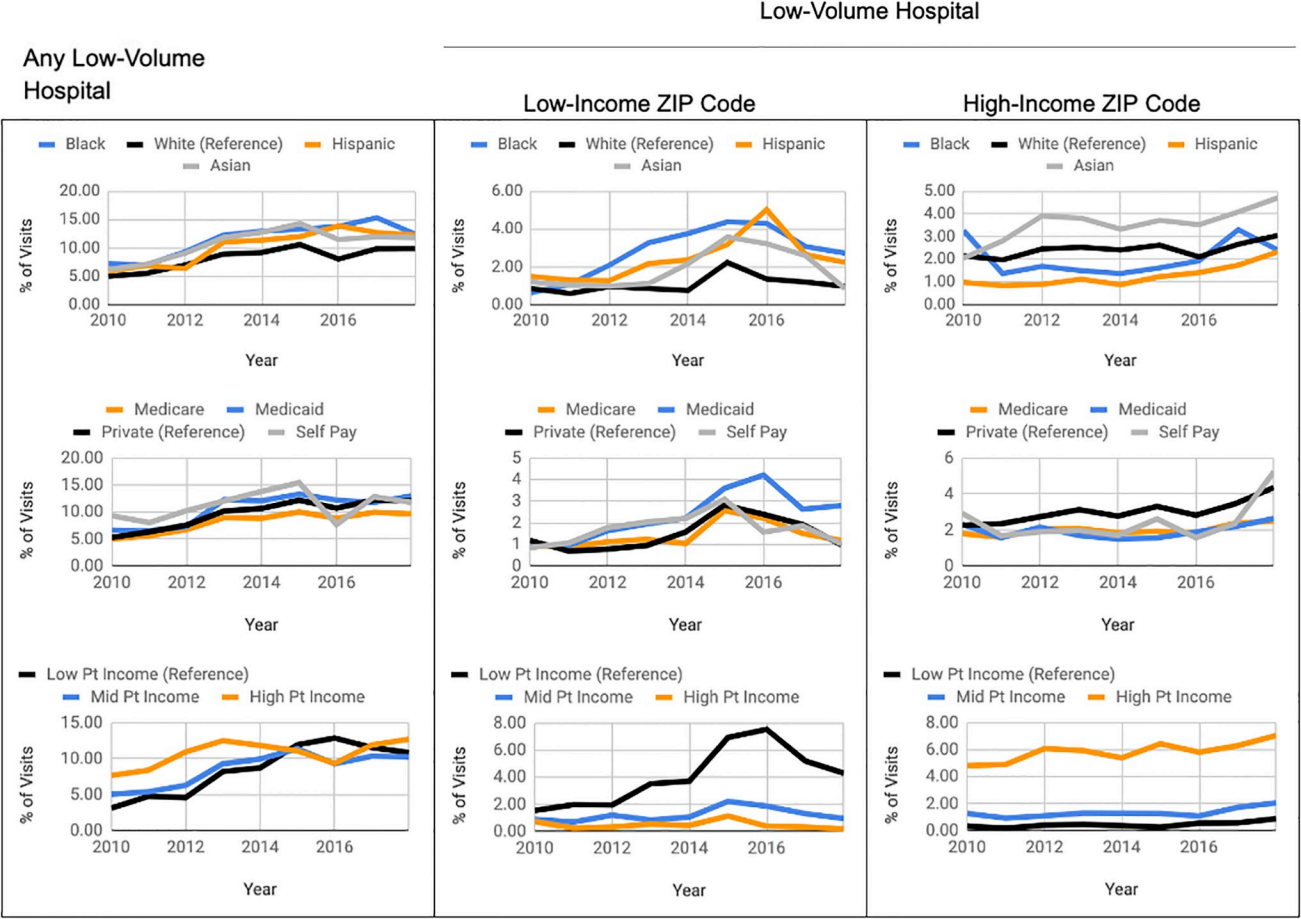
Unadjusted likelihood of receiving PCI at hospitals by volume and income status.

In 2010, Black, Latinx, and Asian patients were more likely to receive PCI at a low-volume hospital compared to non-Latinx White patients. This gap increased across all racial groups in 2018 (Fig 2, left column). The greatest change over time was between Latinx and non-Latinx White patients, where the gap was 166% higher in 2018 (2.4%) compared to 2010 (0.9%).

Considering only hospitals in low-income ZIP codes, in 2010, Latinx and Asian patients were more likely to receive PCI at a low-volume hospital relative to non-Latinx White patients (Fig 2, middle column), in contrast with Black patients (0.6% vs. 0.9% for non-Latinx White patients). In 2018, Black and Latinx patients were more likely to receive PCI in low-volume hospitals relative to non-Latinx White patients, whereas Asian patients were less likely (0.8% vs. 1.0% for non-Latinx White patients). Over the study period, the difference in likelihood of PCI at a low-volume hospital grew the most between Black and non-Latinx White patients (-0.2% in 2010 [e.g., Black patients less likely] to 1.8% in 2018 [e.g., Black patients more likely]).

Considering only hospitals in high-income ZIP codes, in 2010, Black patients were more likely to receive PCI at a low-volume hospital relative to non-Latinx White patients (3.3% vs. 2.1%, respectively; Fig 2, right column), in contrast with Asian and Latinx patients (2.0% and 1.0%, respectively). In 2018, Asian patients were more likely to receive PCI in low-volume hospitals relative to non-Latinx White patients (4.7% vs. 3.0%), in contrast with Black and Latinx patients (2.4% and 2.3%, respectively).

#### By insurance

In 2010, patients with Medicaid or without insurance were more likely to receive PCI at low-volume hospitals compared to patients with private insurance, with differences of 1.4% and 4.0%, respectively (Fig 2, first column). In 2018, the difference decreased to 0.9% between patients with Medicaid and those with private insurance. Patients without insurance were also less likely to receive PCI at low-volume hospitals relative to patients with private insurance (11.8% vs. 12.1%, respectively).

For hospitals in low-income ZIP codes, in 2010, patients with private insurance were more likely to receive PCI at a low-volume hospital (1.2%) vs. 0.9% for patients with Medicare, Medicaid, or no insurance, yielding a gap of 0.3% (Fig 2, middle column). Between patients with Medicaid (2.8%) and patients with private insurance (1.0%), the gap was 500% higher in 2018 compared to 2010.

For hospitals in high-income ZIP codes, in 2010, patients with no insurance were more likely to receive PCI at a low-volume hospital (2.9%) relative to patients with private insurance (2.3%) (Fig 2, right column). This remained true in 2018 (5.2% [uninsured] vs. 4.4% [private pay]) whereas patients with Medicaid and Medicare were less likely (2.6% and 2.5%).

#### By patient income level

Patients with high income were more likely to receive PCI at low-volume hospital than patients with low income. This trend was consistent in 2010 (7.8% vs. 3.1%) and in 2018 (12.7% vs. 10.8%, Fig 2, left column); however, the gap decreased from 4.5% in 2010 to 1.9% in 2018.

Among hospitals in low-income ZIP codes, in 2010, patients with low income were more likely to receive PCI at low-volume hospitals compared to patients with high income (1.6% vs. 0.7%; Fig 2, middle column). In 2018, this gap increased to 4.1%, growing 413%. In contrast, among hospitals in high-income ZIP codes, patients with high income were persistently more likely to receive PCI at low-volume hospitals (4.8% in 2010, 7.1% in 2018) relative to patients with low income (Fig 2, right column).

### Interaction by year

**Fig 3:**
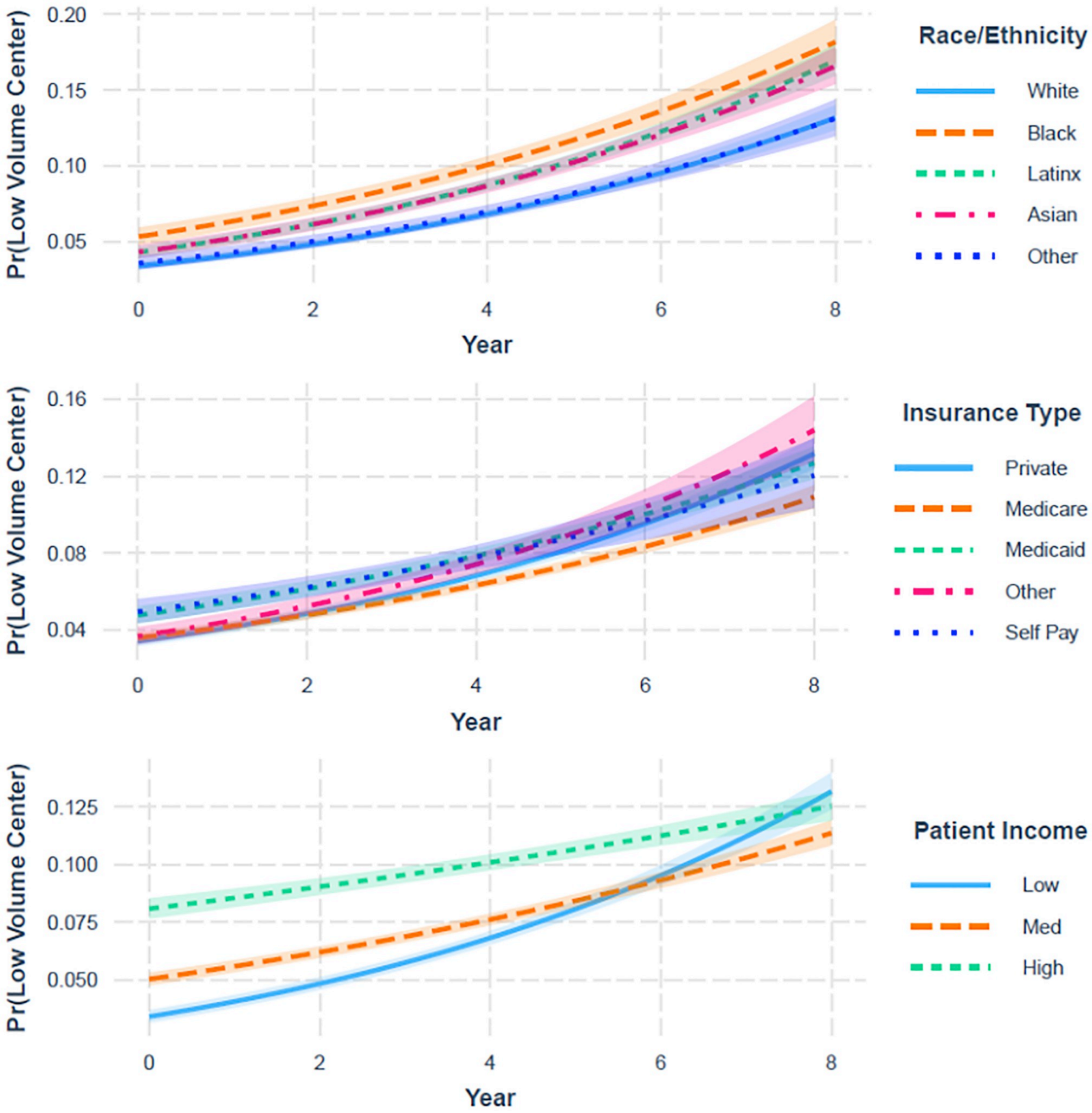
Probability of visiting a low-volume hospital over time by sociodemographic subgroup.

Differences in the relative risk of visiting low-volume PCI hospitals across the study period by race/ethnicity, insurance status, and income group over each year were queried. During the study period, gaps increased between Latinx, Black, and Asian racial/ethnic groups versus non-Latinx White patients (interaction P<0.001, Fig 3). In contrast, the differences in relative risk decreased between patients with non-private insurance and patients with private insurance, and likewise for patients with low income versus high income (interaction P<0.001).

## Discussion

Our study of all hospitalized patients receiving PCI in California hospitals from 2010–2018 revealed that patients from all sociodemographic subgroups were more likely to visit a low-volume PCI hospital over the study period. Additionally, racial/ethnic gaps in the likelihood of receiving PCI at low-volume hospitals were already established in 2010 and were larger in value in 2018. Finally, the racial/ethnic, insurance and income gaps increased in low-income ZIP codes. With regards to our original hypothesis, our study provides mixed results. Black and Latinx patients, as well as patients with Medicaid were more likely to receive PCI at low-volume hospitals relative to their corresponding sociodemographic reference groups. However, compared to patients with low income, patients with high income were more likely to receive PCI at low-volume hospitals, though they were less likely to in low-income neighborhoods.

Our study helps elucidate the potential impact of the decreasing absolute number of inpatient PCIs, increasing number of PCI-capable hospitals, and decreasing rates of MI and coronary artery disease [32–34]. Specifically, all sociodemographic groups experienced an increased likelihood of receiving PCI in a low-volume PCI facility. These are sobering findings given the well-documented relationships between higher volume and improved outcomes [5].

Second, our study suggests a potential mechanism by which racial disparities in cardiovascular outcomes have been persistent [12]. Racial/ethnic minorities are more likely to live in low-income ZIP codes [35], which have been associated with increased emergency medical service times [36, 37], higher overall mortality after cardiac arrest [38], lower rates of revascularization, longer times to the nearest PCI hospital [18], and higher post-acute-MI mortality [39, 40]. In our study, Black, Latinx, Medicaid, and low-income patients were more likely to receive PCI at a low-volume facility in a low-income community, suggesting that poorer quality of care at these hospitals could provide an additional explanation–and potential target of intervention—for racial inequities.

Finally, our study also adds nuance to the role of income in PCI access, showing that receipt of PCI in a low-volume facility is not only increasing for patients who are traditionally underserved. While low-income status is a documented risk factor for poorer PCI access [18], our study showed high-income status as having the highest adjusted relative risk of visits to low-volume hospitals compared to all other sociodemographic factors. Not all visits at low volume hospitals are equal: visits among non-Latinx White patients and patients with high income were predominantly in high-income ZIP codes. This is consistent with previous studies which have suggested that the addition of PCI-capable hospitals have been concentrated in areas with higher market competition and rates of private insurance [41]. Our finding that high income is a risk factor for visiting a low-volume hospital may be driven by known associations between higher income and a higher likelihood of having private insurance, as well as improved access to a higher density of hospitals and physicians [42]. Since there is evidence that new PCI programs were more likely to be started in areas with existing PCI resources, higher income status could therefore be associated with higher PCI resource density areas and thus, lower per-hospital PCI volume [41]. Higher-income communities that are well-served by existing services (or even “over-served”) may experience hospitals with less crowding and may not recognize the potentially detrimental effects of the diffusion of volume across an increasing number of PCI facilities.

Policies that may have contributed to this trend included Senate Bill 891 (2008) and Senate Bill 906 (2015) in California, which amended Section 1256.01 of the Health and Safety Code to increase access to PCI in rural and typically lower-income regions by certifying PCI hospitals without on-site cardiac surgery [43–45]. Paradoxically, these regulations may have also eased burdens for hospitals in suburban areas serving wealthier communities wanting to expand their services for increasing revenue.

Certainly, simple policy recommendations are not easy since some low-volume hospitals improve geographic access, which is necessary for the receipt of timely care known to improve cardiovascular outcomes. Due to the compounding effects of multiple risk factors, there is a need for future research to continue investigating whether PCI outcomes in low-volume hospitals, specifically in low-income ZIP codes, may be worse than in low-volume hospitals in high-income areas. On a population level, the addition of PCI hospitals may drive down per-hospital PCI volume and harm patients differently depending on sociodemographic group [46].

### Limitations

Our study has several limitations. Studies suggest that among patients receiving elective PCIs, there is an increasing proportion of patients receiving outpatient reimbursement and/or same-day discharges, which would not be captured in our inpatient dataset [27]. However, studies that include PCIs from both outpatient and inpatient datasets suggest that prior to 2015, same-day discharge PCIs were relatively infrequent, representing 7.4% in 2013 in one analysis [47], and 6.3% in 2015 in another [48]. Consistent with the methodology in similar studies, we adjusted for expected total PCI volume while operating within the constraints of an inpatient dataset, recognizing that inter-hospital variability in same-day PCIs is not known [29]. Specifically, we observed that studies using the National Cardiovascular Data Registry (NCDR) CathPCI Registry suggest that 60–70% of PCIs are urgent, emergent, or salvage, and an additional one-third of the remaining elective PCIs require inpatient admission due to complications, existing patient comorbidities, and risk factors [27, 29]. One recent analysis using NCDR CathPCI Registry Data reports that for California, 66% of PCIs performed in 2016, 67% in 2017, and 63% in 2018 were non-elective [49–51]. Thus, we estimated that at least 75% of all PCI would qualify as inpatient procedures, using a <150 inpatient PCI volume threshold to approximate the <200 total PCI/year volume referenced in the ACCF/AHA/SCAI guidelines and used by similar PCI volume threshold studies [52]. Finally, approximately 60% of our cohort had a principal diagnosis code of acute MI, consistent with the proportion of emergent PCI reported in studies using the NCDR registry [27, 28], suggesting that inpatient PCI data may still provide a reliable approximation of total PCI volume. Compared to high-volume PCI hospitals, the PCIs performed at low-volume PCI hospitals were more likely to be performed for patients with a principal diagnosis of MI (65.7% vs. 57.0%), suggesting that hospitals with low inpatient PCI volume may have less outpatient PCI volume to add to their total volume compared to hospitals with high inpatient PCI volume.

Second, we used an administrative database that is subject to undercoding, overcoding, or errors in coding. For example, the OSHPD data dictionary categorized all ethnically Latinx patients as also part of a Latinx “normalized racial group,” which this study used, which would under-represent any patients ethnically Latinx but would identify as a separate race. Additionally, the fact that unknown payer and race/ethnicity was categorized as “other” or omitted in our study may systematically undercount minorized race/ethnicity or insurance groups. We used ICD-9 and ICD-10 codes modeled after similar analyses of PCI utilization from inpatient datasets [20, 22], reducing the likelihood of systematic error in selecting the population of interest. This dataset is also a well-known source, relied upon by thousands of studies, and specifically it has also been used for similar analyses of trends in PCI [53, 54].

Third, while the clinical significance of any PCI volume threshold is still debated, there are several recent studies demonstrating the association of lower volumes to higher rates of inpatient mortality and post-procedural complications [54, 55]. Some of the increase in inpatient mortality found in these studies may be explained by the removal of uncomplicated PCI from the denominator as more patients are considered eligible for “outpatient” PCI. We did not choose to evaluate the quality of hospitals through alternate methods such as using ICD-9 and ICD-10 codes to capture the common complications of PCI performed in low- versus higher-volume hospitals, in part because of the limitations of the administrative databases stated above. Additionally, total PCI volume remains an important value of measurement in that it is still used for thresholds in guidelines [5] and PCI hospital certification [6, 7], and it is thus of clinical relevance.

Another limitation was that the operator volumes were not analyzed. For example, if high-volume operators traveled and worked at low-volume hospitals, this study may underappreciate the potential good outcomes received by patient subgroups that were more likely to receive PCI at low-volume hospitals in this study.

Finally, our study is limited to California, which has been shown to have a PCI density that is below the national median; thus, California may face unique trends and challenges not generalizable to the entire United States [56].

## Conclusion

We report an increased likelihood of visits at low-volume PCI hospitals across all sociodemographic groups, including White and high-income patients as well as traditionally underserved groups. Black, Latinx, and Asian patients faced a higher likelihood of receiving PCI in a low-volume hospital relative to non-Latinx White. These differences were accentuated when examining receipt of PCI in low-volume hospitals in low-income communities, where Medicaid and low-income patients also had a differentially higher increase compared with privately insured and high-income patients, respectively. In high-income communities, these sociodemographic differences were attenuated or reversed.

## Supporting information

S1 Table PCI ICD-9-CM procedure codes. ICD-9-CM International Classification of Diseases 9th Revision, Clinical Modification.
(DOCX)

S2 Table PCI ICD-10 PCS codes. ICD-10-PCS International Classification of Diseases 10th Revision, Procedural Classification System.
(DOCX)

S3 Table ICD-9 and ICD-10 codes for comorbidities. CHF—congestive heart failure; CKD—chronic kidney disease; ICD—International Classification of Diseases; MI—Myocardial infarction; ICD-9 International Classification of Diseases 9th Revision; ICD-10 International Classification of Diseases 10th Revision.
(DOCX)
