# Supplementary material for: Trends in the likelihood of receiving percutaneous coronary intervention in a low-volume hospital and disparities by sociodemographic communities
Source: PLoS One. 2023 Jan 18;18(1):e0279905. doi: 10.1371/journal.pone.0279905 (PMC9847957; doi:10.1371/journal.pone.0279905)
Supplement: S1 Table — ICD-9-CM International Classification of Diseases 9th Revision, Clinical Modification. (DOCX) [file pone.0279905.s001.docx]

| **Table S1. PCI ICD-9-CM Procedure Codes** | |
| --- | --- |
| ICD-9-CM Procedure Code | Description |
| 17.55 | Transluminal coronary atherectomy |
| 00.66, 36.01, 36.02, 36.05 | Percutaneous transluminal coronary angioplasty |
| 36.06 | Insertion of non-drug-eluting coronary artery stent(s) |
| 36.07, 36.09 | Insertion of drug-eluting coronary artery stent(s) |

ICD-9-CM International Classification of Diseases 9th Revision, Clinical Modification
