# Supplementary material for: Trends in the likelihood of receiving percutaneous coronary intervention in a low-volume hospital and disparities by sociodemographic communities
Source: PLoS One. 2023 Jan 18;18(1):e0279905. doi: 10.1371/journal.pone.0279905 (PMC9847957; doi:10.1371/journal.pone.0279905)
Supplement: S2 Table — ICD-10-PCS International Classification of Diseases 10th Revision, Procedural Classification System. (DOCX) [file pone.0279905.s002.docx]

| **Table S2. PCI ICD-10 PCS Codes** | |
| --- | --- |
| ICD-10-PCS Code | Description |
| 02703xx | Percutaneous Coronary Artery Dilation, One Artery |
| 02704xx | Percutaneous Endoscopic Coronary Artery Dilation, One Artery |
| 02713xx | Percutaneous Coronary Artery Dilation, Two Arteries |
| 02714xx | Percutaneous Endoscopic Coronary Artery Dilation, Two Arteries |
| 02723xx | Percutaneous Coronary Artery Dilation, Three Arteries |
| 02724xx | Percutaneous Endoscopic Coronary Artery Dilation, Three Arteries |
| 02733xx | Percutaneous Coronary Artery Dilation, Four or More Arteries |
| 02734xx | Percutaneous Endoscopic Coronary Artery Dilation, Four or More Arteries |

ICD-10-PCS International Classification of Diseases 10th Revision, Procedural Classification System
