# Supplementary material for: Trends in the likelihood of receiving percutaneous coronary intervention in a low-volume hospital and disparities by sociodemographic communities
Source: PLoS One. 2023 Jan 18;18(1):e0279905. doi: 10.1371/journal.pone.0279905 (PMC9847957; doi:10.1371/journal.pone.0279905)
Supplement: S3 Table — CHF—congestive heart failure; CKD—chronic kidney disease; ICD—International Classification of Diseases; MI—Myocardial infarction; ICD-9 International Classification of Diseases 9th Revision; ICD-10 International Classification of Diseases 10th Revision. (DOCX) [file pone.0279905.s003.docx]

| **Table S3. ICD-9 and ICD-10 Codes for Comorbidities** | | |
| --- | --- | --- |
| Comorbidity | ICD-9 Code | ICD-10 Code |
| Acute MI | 410.x0, 410.x1 | I21.x |
| Cardiogenic shock or cardiac arrest | 785.51, 427.5 | R57.0, I46.9 |
| CHF | 428-428.9 | I50.x |
| Cerebrovascular accident | 433.x, 434.x | I63, I65, I66 |
| Diabetes | 250-250.3, 250.7 | E08-E13 |
| HTN | 401-405 | I10.x |
| CKD | 585.x | N18.x |

CHF - congestive heart failure; CKD - chronic kidney disease; HTN - hypertension; ICD - International Classification of Diseases; MI - Myocardial infarction; ICD-9 International Classification of Diseases 9th Revision; ICD-10 International Classification of Diseases 10th Revision
